# Convolutional neural network-based reconstruction for positronium annihilation localization

**DOI:** 10.1038/s41598-022-11972-5

**Published:** 2022-05-20

**Authors:** Jin Jegal, Dongwoo Jeong, Eun-Suk Seo, HyeoungWoo Park, Hongjoo Kim

**Affiliations:** 1 Department of Physics, Kyungpook National University, Daegu, 41566 Korea; 2 Department of Physics, University of Maryland, College Park, MD 20742 USA

**Keywords:** Experimental particle physics, Characterization and analytical techniques

## Abstract

A novel hermetic detector composed of 200 bismuth germanium oxide crystal scintillators and 393 channel silicon photomultipliers has been developed for positronium (Ps) annihilation studies. This compact 4π detector is capable of simultaneously detecting γ-ray decay in all directions, enabling not only the study of visible and invisible exotic decay processes but also tumor localization in positron emission tomography for small animals. In this study, we investigate the use of a convolutional neural network (CNN) for the localization of Ps annihilation synonymous with tumor localization. Two-γ decay systems of the Ps annihilation from ^22^Na and ^18^F radioactive sources are simulated using a GEANT4 simulation. The simulated datasets are preprocessed by applying energy cutoffs. The spatial error in the XY plane from the CNN is compared to that from the classical weighted k-means algorithm centroiding, and the feasibility of CNN-based Ps annihilation reconstruction with tumor localization is discussed.

## Introduction

A positronium (Ps) is a quasi-stable bound system of an electron and its anti-particle, a positron. The two particles approach closer and closer to each other, turning into γ-rays until they finally annihilate one another. Ps annihilation is not only used for fundamental physical research, such as standard model verification^1^ and new physics model discovery^2^, but also applied research, such as Ps annihilation lifetime spectroscopy (PALS)^3,4^ and positron emission tomography (PET)^5^. In these studies, Ps annihilation localization is one of the important factors because since Ps annihilation cannot be directly measured, in most cases, the annihilation position can only be reconstructed through the measurement of subsequent γ-ray energy. Therefore, great effort is required to arbitrarily control the annihilation position or to reconstruct based on the subsequent measurement.

Recently, convolutional neural network (CNN)-based data reconstruction has shown especially successful performance when utilized for charged particle tracking with good precision in accelerator and calorimeter experiments^6–8^. Additionally, CNNs have effectively overcome the spatial resolution limitations of PET, which is essentially limited by the size of the detector array elements, such as the crystal scintillator and readout pixels used in medical imaging^9^. These high-performance results can be obtained through Monte Carlo (MC) simulations, which can generate sufficient training data and represent the geometry of the detector well. Consequently, CNNs are expected to be suitable for the reconstruction of Ps annihilation and background noise cutoff at the Kyungpook National University Advanced Positronium Annihilation Experiment (KAPAE) detector.

The KAPAE detector, which is a 4π detector, comprises 200 Bi_4_Ge_3_O_12_ (BGO) crystal scintillators and 393 channels of silicon photomultipliers (SiPMs). In the KAPAE detector, a ^22^Na radioactive source is used to generate positrons from β+ decay. The instrument configuration is optimized to trigger on positrons by varying the polyethylene naphthalate (PEN) film plastic scintillator thickness^10^. The KAPAE detector aims to study CPT violation in Ps annihilation physics^10,11^. Based on the relative spin orientations, the Ps ground state has two possible configurations: the triplet state (^3^S_1_), or ortho positronium (o-Ps), and the singlet state (^1^S_0_), or para positronium (p-Ps). Due to C-parity conservation, p-Ps and o-Ps decay to even and odd numbers of photons, respectively. Since these processes possess different C-parity values, the precise distinction of p-Ps and o-Ps is important to test discrete symmetries of C, CP, and CPT in the lepton sector^12^.

In this study, data reconstruction based on a CNN focusing on a back-to-back 2-γ decay system is conducted. The 2-γ energies are deposited in the surrounding BGO scintillators, and this process is simulated using the GEANT4 simulation toolkit^13^. The simulation data are used to produce datasets for reconstructions based on the CNN and weighted k-means algorithm^14^. The k-means clustering algorithm is a conventional method to typically determine the clustering centroid for uncategorical datasets, and it has been utilized in the abovementioned fields of CNN applications. Through this 2-γ decay system data reconstruction, we can distinguish the p-Ps signal from the o-Ps signal for the background noise cutoff and detect o-Ps events more correctly with high efficiency. Additionally, note that the size of the KAPAE detector is compact (150 × 150 × 150 mm^3^), and it can simultaneously detect γ-ray decays in all directions. This feature makes it possible to utilize the KAPAE detector with an ^18^F radioactive source in PET application for small animal tumor localization^15^.

## Results

### Energy cutoff criterion

**Figure 1:**
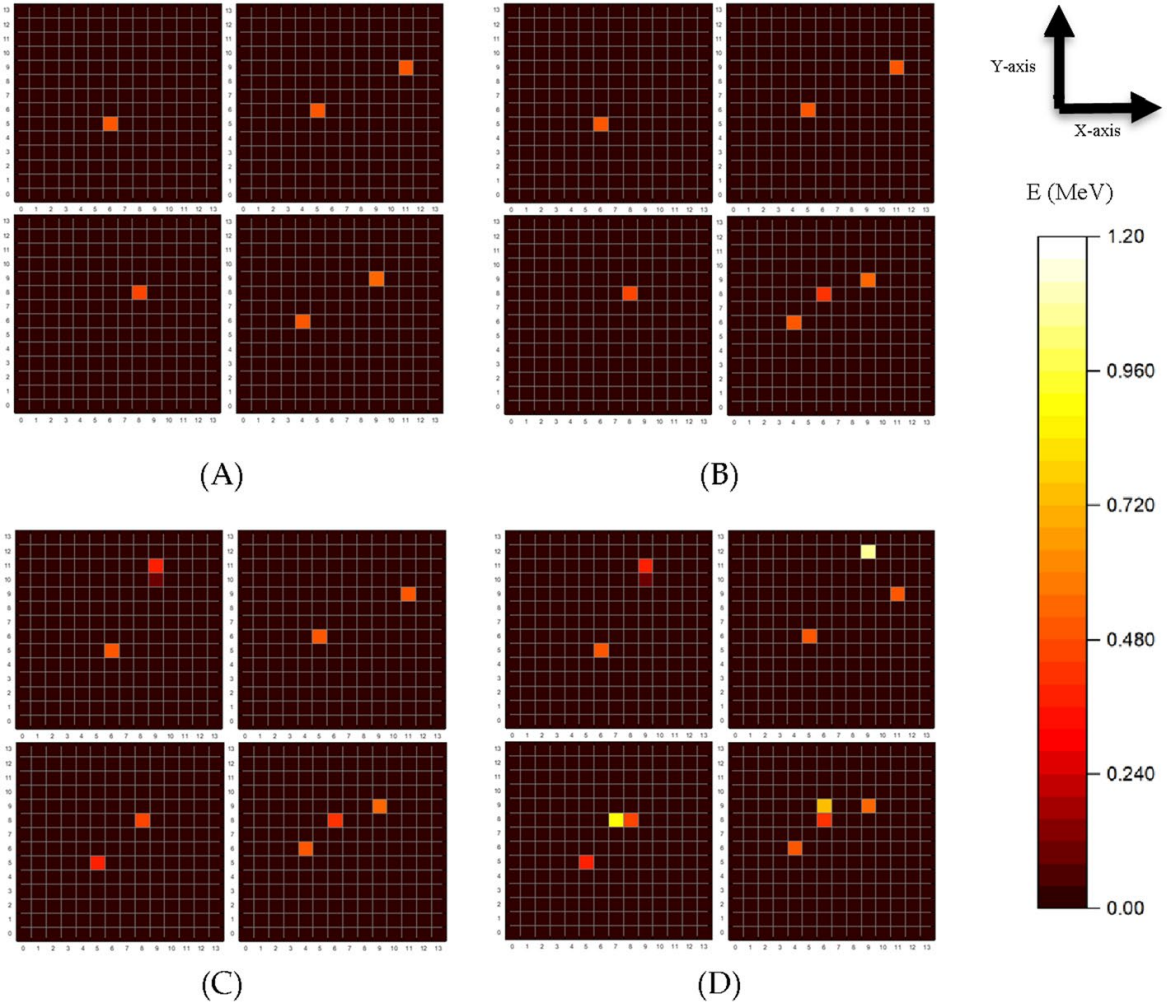
The γ energy distribution of a top view of the BGO scintillators. An example of four randomly selected events are compared for the four different energy cutoff ranges given in Table 1: (**A**) 1σ, (**B**) 2σ, (**C**) above 2σ, and (**D**) no cutoff.

Four sets of data corresponding to various energy cuts centered at 511 keV are used to train the data. In Table 1, “1σ” and “2σ” are the energy cut between ± 1σ and ± 2σ centered at 511 keV, and “ > 2σ” is the energy cutoff ranges of more than 2σ, 0.6 MeV. The number of events varies corresponding to the energy cutoff ranges, as shown in Fig. 1. However, as we maintain the batch size to 3% of the dataset for the same number of interactions in each case, these four sets can be compared to determine how different energy cut ranges affect the reconstruction.

**Table 1:** XY RMSE [mm] of Ps annihilation localization using CNN-based reconstruction depending on the energy cutoff criteria.

Radioactive source	1σ	2σ	> 2σ	Not applied
^22^Na	4.69	4.52	4.19	4.17
^18^F	4.53	4.33	3.93	3.88

**Figure 2:**
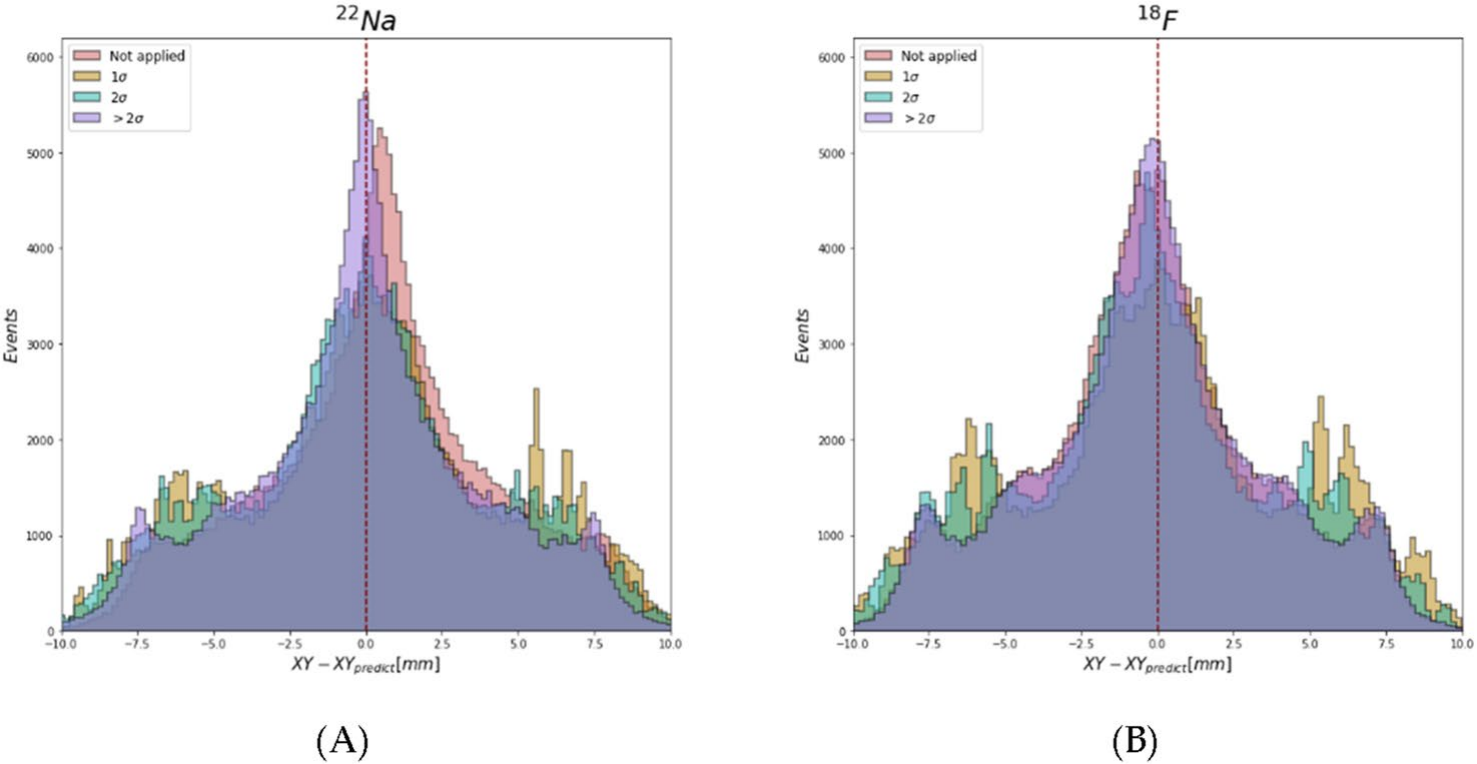
Difference between reconstructed and initial Ps annihilation positions corresponding to the energy cutoff range of (**A**) ^22^Na and (**B**) ^18^F.

The root mean square error (RMSE) of Ps annihilation localization using CNN depending on the energy cut criterion and radioactive sources is summarized in Table 1. The RMSE is calculated by comparing the XY coordinates of Ps in each event from the GEANT4 simulation (initial) with the reconstruction position based on the CNN (predicted). In the test data, 100,000 events are used for calculating the RMSE. Since the X and Y coordinates are not correlated, the RMSE is calculated all at once. As shown in Table 1, when σ is increased to include the low energy contribution from Compton scattering, the spatial error in the XY plane decreases. In addition, when 1.28 MeV γ-rays from the ^22^Na source is included, the RMSE is further decreased. However, the reconstructed position is closer to the initial value for the smaller σ cases where 1.28 MeV γ-ray events are not included, as shown in Fig. 2. This means that the inclusion of the correlated information in the 2-γ decay is helpful for training the CNN model. For the 1σ and 2σ cases, there are peaks between ± 5.0 mm and ± 7.5 mm, resulting in lower accuracy than other cases. Therefore, the optimized energy cutoff criterion for the back-to-back 2-γ decay system discrimination modeling is determined as “ > 2σ”, where the spatial error in XY is 4.19 mm for ^22^Na and 3.93 mm for ^18^F.

### The CNN performance

The Ps annihilation localization performance of the CNN is compared with that of classical centroiding weighted k-means algorithm. The “ > 2σ” energy cutoff is used to achieve the best accuracy in the XY plane. The RMSE between the simulated XY position of Ps annihilation and that predicted by CNN is calculated. It is compared with the RMSE for the weighted k-means clustering algorithm in Table 2. The RMSE of the CNN with our proposed architecture is 2.2 times smaller than that of the weighted k-means clustering algorithm.

**Table 2:** The RMSE [mm] of Ps localization based on the weighted k-means algorithm and the CNN-based reconstruction corresponding to the radioactive source.

Radioactive source	Weighted k-means algorithm	The CNN
^22^Na	9.42	4.19
^18^F	8.49	3.93

**Figure 3:**
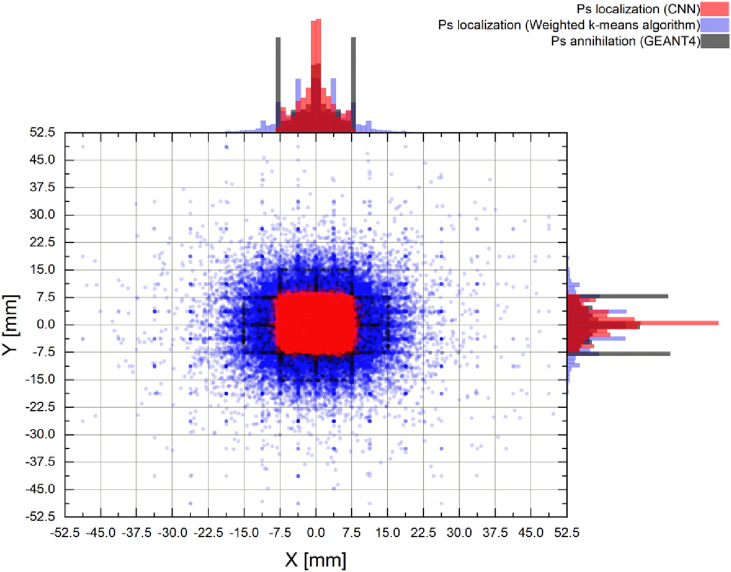
The obtained Ps annihilation localizations for the case of ^18^F using the CNN and the weighted k-means algorithm; the XY coordinates are for the top view of the KAPAE detector.

**Figure 4:**
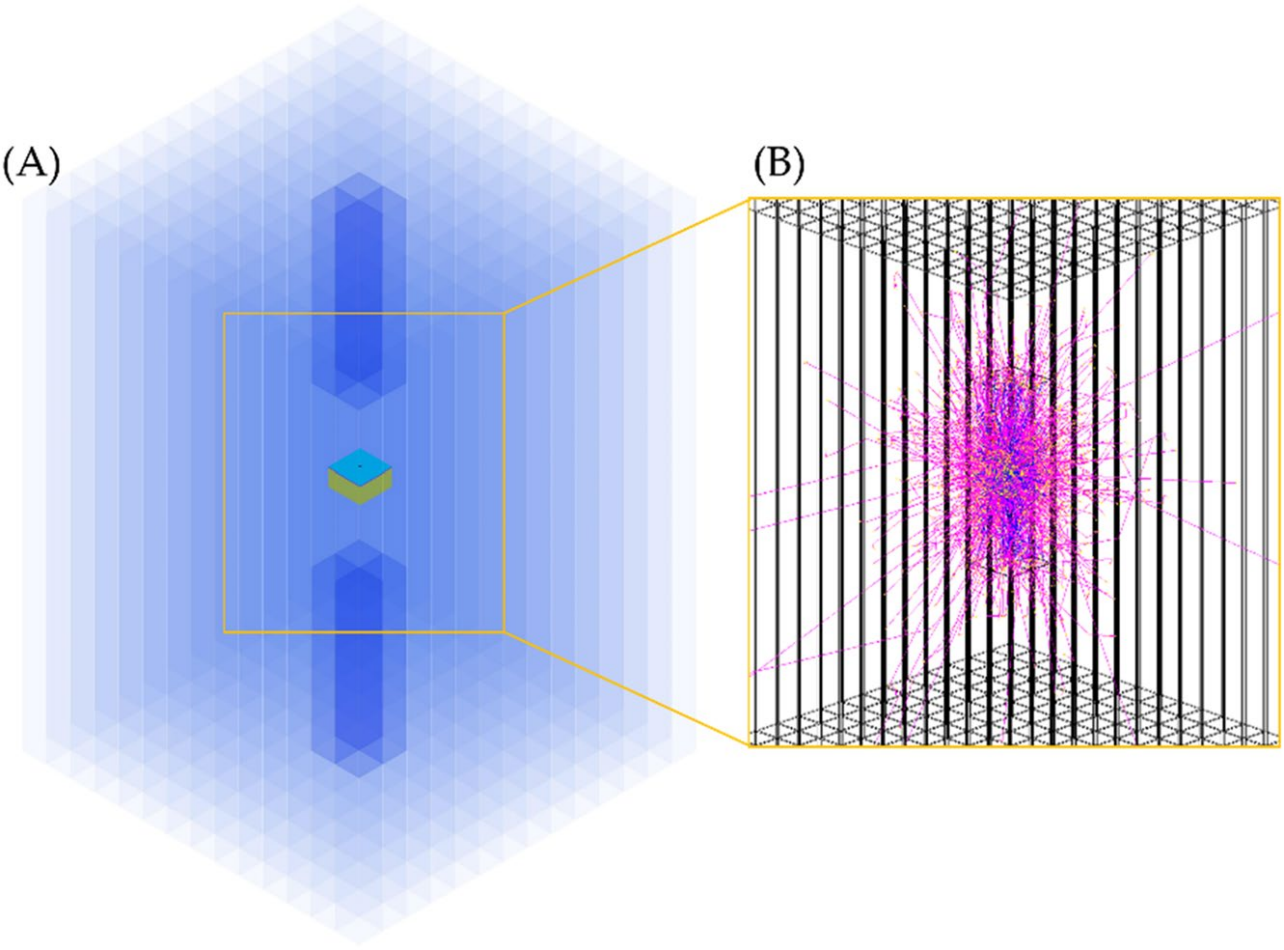
(**A**) The simulation geometry of the KAPAE detector excluding SiPMs. BGO scintillator bars are shown in blue. The yellow box with a cyan top at the center of the KAPAE detector represents the trigger system including the radioactive source. (**B**) A simulated 2-γ decay system. Red lines represent tracks of charged particles produced from interactions of γ-rays with the BGO scintillators.

Figure 3 shows the Ps annihilation localization of the CNN and weighted k-means clustering; they are compared with the initial position from the GEANT4 simulation data in the case of ^18^F. In the GEANT4 simulation data, most positrons are generated from the β + decay of a radioactive source. Since the radioactive source is located at the center of the trigger system, (0,0) mm in the XY plane, most of the Ps annihilate in the trigger space within ± 7.5 mm (XY) (Fig. 4). In addition, an increase in the number of Ps annihilations at the edge of the trigger space near ± 7.5 mm (XY) is due to positron diffraction in the solid BGO crystal^16^. The weighted k-means algorithm is repeatedly trained to determine the clustering centroid until the distance between the centroid and its adjacent components is minimized. In contrast, the CNN is optimized to directly minimize the error by comparing the initial position data until the results converge to a minimum loss index. The CNN can adapt to various circumstances even with irregular dataset information by updating weight. Consequently, the CNN results (Fig. 3) show that the reconstructed Ps annihilation localization is more accurate than the weighted k-means clustering-based localization.

## Discussion

The classical method of reconstructing Ps annihilation sites has become saturated in terms of PET medical imaging. In recent years, the PET/CT fusion equipment has been developed^17^, and depth-of-interaction encoding technology has been applied to the time-of-flight PET^18^. To dramatically overcome these technical limitations, the application of deep learning is a natural procedure. In particular, an image-optimized CNN is the best candidate method. Studies starting from this point of view had data predate processing, overfitting, and errors in the interpretation of learning results. This may be an error in the interpretation of the result because the principle of the detector is not understood. We conducted a study with a high degree of understanding of the learning results using a self-developed KAPAE detector as a model.

Finally, this study performed CNN-based Ps annihilation reconstruction using the KAPAE detector and compared the results with a conventional weighted k-means algorithm. The detector geometry and the back-to-back 2-γ decay system produced through p-Ps annihilation are simulated using the GEANT4 simulation, which generates enough data for training. ^22^Na and ^18^F radioactive sources are used for CPT violation study and PET application, respectively. The energy cutoff criterion of over 2σ is determined using the proposed CNN architecture and by comparing the RMSEs in each radioactive source. For ^22^Na, using the weighted k-means algorithm and the CNN, Ps annihilation is reconstructed with RMSEs of 9.42 mm and 4.19 mm, respectively. For ^18^F, using the weighted k-means algorithm and the CNN, Ps annihilation is reconstructed with RMSEs of 8.49 and 3.93 mm, respectively. ^22^Na has a relatively long-term half-life compared to ^18^F and is used for rare decay or CPT tests, which require several months to several years of measurement time. ^18^F has a short half-life, emits γ-ryas in large amounts in several minutes and is useful for diagnostic imaging and techniques. Comparing the results of the weighted k-means algorithm and the CNN together, the RMSE of ^18^F is smaller. This is due to the absence of 1.28 MeV γ-rays in the ^22^Na decay, which shows that ^18^F can be trained well without preprocessing by applying energy cutoffs and is advantageous for PET application when reconstructing Ps annihilation localization compared to ^22^Na. In conclusion, the proposed CNN architecture achieved approximately two times better spatial resolution in the XY plane compared to the weighted k-means algorithm. Thus, the proposed CNN architecture can be applied to distinguish p-Ps from o-Ps for CPT violation studies from subsequent γ energy deposited on BGO scintillators as well as to localize the tumor position in PET for small animals.

## Methods

### Monte Carlo simulation

Figure 4 shows the GEANT4 simulation of the radioactive decay from a ^22^Na or ^18^F point source in the KAPAE detector. The simulated detector comprises 192 BGO scintillators with dimensions of 7.5 × 7.5 × 150 mm^3^ and 8 endcap BGO scintillators with dimensions of 7.5 × 7.5 × 50 mm^3^ surrounding the trigger system. Each scintillator is covered by a VM2000 reflector of 75 μm thickness. A point source is placed at the center of the PEN film plastic scintillator, represented by the yellow box in the middle of Fig. 4. The KAPAE detector is filled with nitrogen gas, and a silica aerogel is used for the generation of o-Ps annihilation for minimization of a pick-off effect^10^. We simulate the 2-γ system of p-Ps signals only because we can discriminate o-Ps in real data by developing a p-Ps data reconstruction model. The real data for the γ energy spectrum from the BGO scintillator are colleted using SiPMs attached to both ends of the KAPAE detector. Instead of replicating all SiPMs in the simulation, only the SiPM photon detection efficiency from the experiment is considered in the simulation.

**Figure 5:**
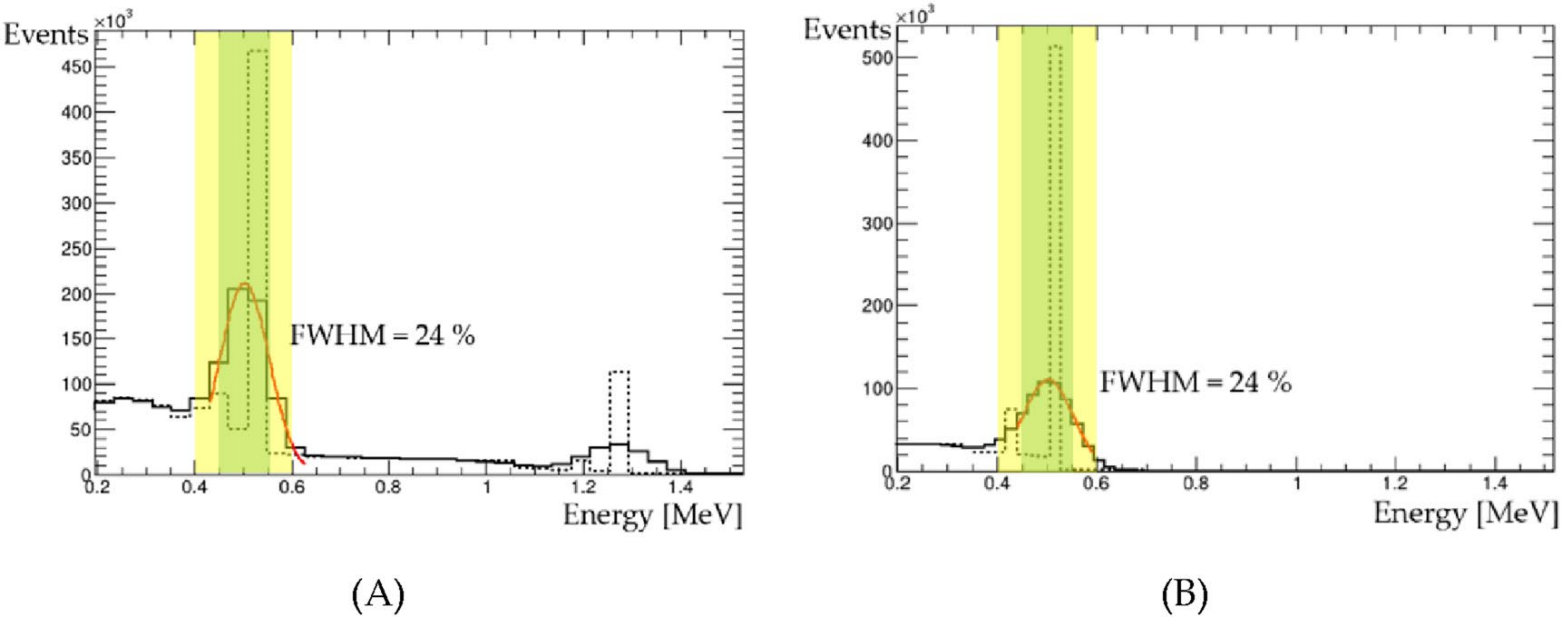
Energy spectra of 511 keV γ-rays from the (**A**) ^22^Na source and (**B**) ^18^F source simulated using a BGO scintillator in the KAPAE detector. The solid line considers the statistical noise fluctuation, but the dotted line does not. The yellow shade denotes the 2σ range, and the green box denotes the 1σ range. The red line is a Gaussian fit to the data.

One million events of the 2-γ decay system of p-Ps are initially generated for discrimination with o-Ps in real data, and only events passing through the trigger system are selected. The pseudo data are processed using the scintillation energy resolution in the BGO scintillator scintillation from a preliminary experiment utilizing the SiPMs. The total statistical noise fluctuation (σ/E) is proportional to (1/$\sqrt{\mathrm{n}}$), given by the Poisson distribution as follows:

1 $$\frac{\sigma}{E} = k\frac{1}{\sqrt{n}}$$

where k is the proportional constant, n = L × E × PDE is the number of photons, L is the absolute light yield of the scintillator (photons/MeV), E is the energy (MeV), and PDE is the scintillation light detection efficiency of the SiPM. The pseudo data of ^22^Na and ^18^F are processed using Eq. (1). The energy spectra of 511 keV γ-rays from both sources are shown in Fig. 5. The full width at half maximum (FWHM) is 24% for each source.

The pseudo data are written in Python for the machine learning framework and processed to a matrix of pixels corresponding to the BGO scintillator array position mapping. The data are restructured to a 14 × 14 matrix for the top view of the detector. The central 4 × 4 data are from the deposited energy of the short 8 endcap BGO scintillators. The 4 endcap scintillators above the trigger system are paired with the 4 endcap scintillators below the trigger system. The energy deposit sum of each pair forms the central 4 × 4 data. Four sets of data corresponding to various energy cuts centered at 511 keV are used to train the data (Table 3).

**Table 3:** The number of events corresponding to the energy cutoff ranges.

Radioactive source	1σ	2σ	> 2σ	Not applied
^22^Na	394,839	467,378	520,228	536,256
^18^F	427,965	506,384	549,850	550,540

### Weighted K-means algorithm

To evaluate the CNN performance, the conventional weighted k-means algorithm^19^ is also utilized. The k-means algorithm employs an iterative approach to group the data into k predetermined clusters by minimizing the sum of squared errors (SSE). The SSE is obtained as follows:

2 $${\text {SSE}} = \mathop \sum \limits_{{i = 1}}^{n} \mathop \sum \limits_{{j = 1}}^{k} w^{{(i,j)}} \left\| {x^{{(i)}} - \mu ^{{(j)}} } \right\|^{q}$$

where μ^(j)^ is the centroid of the jth cluster, x^(i)^ is the data sample, k is the number of clusters, n is the number of elements in the dataset, and q is an integer that defines the nature of the distance function (q is 2 for the Euclidean distance). Furthermore, μ^(j)^ is 1 if the data sample x^(i)^ belongs to the jth cluster and 0 otherwise. The weighted k-means algorithm is utilized in the scikit-learn package. Unlike the CNN data, the center XY position of the BGO scintillator deposited with γ energy is used as the data. Each position has a weight corresponding to the deposited γ energy of the BGO scintillator. Since a radioactive source is centered on the detector, k is set to 1, and the Ps annihilation position will be biased by the γ energy.

### CNN

**Figure 6:**
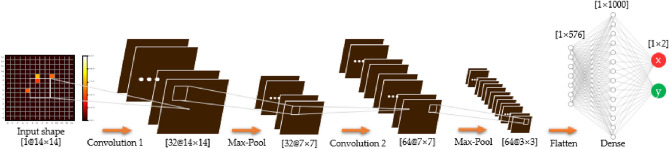
The proposed CNN architecture for Ps annihilation localization in the KAPAE detector.

- CNN architecture
  The proposed CNN architecture is initialized using Keras 2.1.6 with TensorFlow 2.4.0 as a backend in Python 3.8.5^20^. The network architecture (Fig. 6) comprises two convolutional layers as feature extraction and the output dense layer. Various architectures are set up and tested, and the number of convolutional, pooling, and fully connected layers and the number of filters in each layer are determined. Starting with a 14 × 14 input shape, rectified linear unit (ReLU) activation, defined as f(x) = max(x, 0)^21^, is performed for each functional layer, including the dense layer. For regression, a linear activation is performed at the end of the dense layer to output the 2D coordinates (XY) of the Ps annihilation position as a 1 × 2 vector.
- Model training and testing

MC simulation data of the 2-γ decay of the ^22^Na and ^18^F radioactive sources are employed for the training. By utilizing the GEANT4 simulation and excluding the empty matrix data where all components are 0, sufficient events are generated to train the CNN. The CNN training is performed by using 70% of the dataset as a training set and 30% of the training set is used for training validation. The training set is used for CNN architecture and hyper-parameter optimization. As an optimizer, Nesterov-accelerated adaptive moment estimation (Nadam)^22^ is used for training optimization with an initial learning rate of 0.001. Nadam is an advanced optimizer with a Nesterov-accelerated gradient added to the adaptive moment estimation (Adam)^23^. Moreover, it can advantageously find the global minimum more quickly and accurately than Adam by determining the gradient after moving to the momentum value rather than determining the gradient and momentum values to move from the current position to the next position^24^. The mean squared error (MSE) is used as the loss metric. The batch size is adapted to maintain the batch size as 3% of the dataset, and the number of epochs is optimized to the epoch just before overfitting occurs. Then, the model converges in the direction of minimizing the MSE and the mean absolute error as a function of the number of epochs. These loss curves reach approximately equal minimums with asymptotic behavior. The remaining 30% of the dataset is used for testing the model.

## Supplementary Information

Supplementary Video 1.

﻿Supplementary Information 1.
