# Supplementary material for: Convolutional neural network-based reconstruction for positronium annihilation localization
Source: Sci Rep. 2022 May 20;12:8531. doi: 10.1038/s41598-022-11972-5 (PMC9122910; doi:10.1038/s41598-022-11972-5)
Supplement: Supplementary file 2 — ﻿Supplementary Information 1. [file 41598_2022_11972_MOESM2_ESM.pdf]

# <The additional comparative methods>

- **For asserting the outstanding performance of the CNN, we also make a comparative study with the Y network and Resnet methods.**

Table 1. The RMSE [mm] of Ps localization based on the Y-network and the Resnet (depth =8) corresponding to the radioactive source.

| Radioactive source | Y-network | Resnet (depth =8) |
|---|---|---|
| $^{22}Na$ | 4.2 | 4.3 |
| $^{18}F$ | 4.0 | 4.0 |

- The architectures

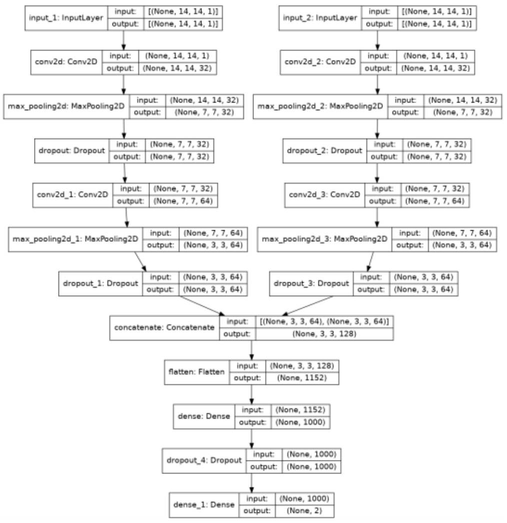


[The architecture of Y-network]

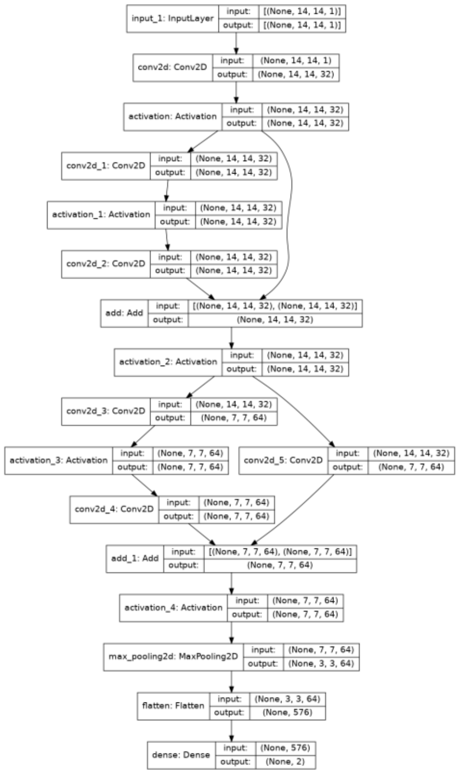


[The architecture of Resnet (8)]
